# Anemia of cGKI deficient mice is caused by intestinal bleeding

**DOI:** 10.1186/2050-6511-16-S1-A35

**Published:** 2015-09-02

**Authors:** Elisabeth Angermeier, Katrin Domes, Franz Hofmann

**Affiliations:** 1 Forschergruppe 923, Institut für Pharmakologie und Toxikologie, Technische Universität München, Munich, Germany

## Background

Inactivation of the cGKI gene is associated with anemia in mice [1]. It has been shown that cGKI-/- and cGKI rescue mice develop a bleeding duodenal ulcer [2]. We analysed the cause for this anemia.

## Results

cGKI-/- and cGKI rescue mice have significant lower erythrocyte levels than their control littermates, lower hematocrit and hemoglobin concentrations and elevated reticulocyte numbers. The spleens of these mice are massively enlarged. cGKI mutant mice clearly suffer from iron deficiency due to decreased plasma iron levels and the lack of iron in the spleen and liver. To maximize intestinal iron absorption in cGKI -/- mice hepcidin mRNA levels are down regulated in the liver. The iron regulatory hormone hepcidin binds to the only known cellular iron exporter ferroportin which leads to its degradation and therefore inhibits the iron transport via ferroportin [3,4]. IL-6 which is able to modulate the expression of hepcidin was elevated in the serum of cGKI mutants compared to controls [5].

Feeding the proton pump inhibitor (PPI) esomeprazol (18 mg/kg chow) did stop the intestinal bleeding as shown by Haemoccult test and prolong the survival of cGKI-/- mice. Perls Prussian blue tissue staining for ferric iron showed that PPI treatment rescues the iron deficiency in the spleen of cGKI -/- mice and normalized serum iron levels. These results were confirmed by western blot analysis for the iron storage protein ferritin light chain. In the liver ferritin levels and hepcidin mRNA levels are increased. Treatment with PPI normalizes red blood cell counts, hematocrit and hemoglobin levels as well as reticulocyte numbers and prevents splenomegaly. Serum IL-6 concentration was not affected by PPI treatment of cGKI mutants.

## Conclusion

These data indicate that anemia and splengomegaly in cGKI-/- and cGKI rescue mice is caused by iron deficiency anemia due to intestinal blood loss and can be rescued by treatment with esomeprazol.
